# Consumer Knowledge about Dietary Relevance of Fruits and Vegetables: A Study Involving Participants from Portugal and France

**DOI:** 10.3390/nu16020287

**Published:** 2024-01-18

**Authors:** Nolwenn Le Turc, Ana Jaquelina Silva, Sofia G. Florença, António Raposo, João Carlos Gonçalves, Maria João Lima, Edite Teixeira-Lemos, Raquel P. F. Guiné

**Affiliations:** 1 Department of Food Industry, Agrarian School of Viseu, 3500-606 Viseu, Portugal; nleturc44@gmail.com (N.L.T.); silva17jaque@gmail.com (A.J.S.); jgoncalves@esav.ipv.pt (J.C.G.); mjoaolima@esav.ipv.pt (M.J.L.); raquelguine@esav.ipv.pt (R.P.F.G.); 2 IUT University Institute of Technology, Angers University, 49035 Angers, France; 3 CERNAS-IPV Research Centre, Polytechnic University of Viseu, 3504-510 Viseu, Portugal; sofiaflorenca@outlook.com; 4 CBIOS (Research Center for Biosciences and Health Technologies), Universidade Lusófona de Humanidades e Tecnologias, Campo Grande 376, 1749-024 Lisboa, Portugal

**Keywords:** healthy diet, fruit consumption, vegetable consumption, factor analysis, cluster analysis

## Abstract

Fruits and vegetables are recommended as low-calorie foods that contribute to the proper intake of necessary micronutrients, macronutrients, and bioactive compounds with health benefits. However, the recommendations for the dietary intake of these foods fail to be attained in most European countries. For this reason, promoting more knowledge about the health effects of fruits and vegetables is essential to decrease the incidence of chronic diseases. This study was conducted to investigate the knowledge of the health benefits of fruits and vegetables among the population of Portugal and France. The present work involved a questionnaire survey of 639 participants (257 from Portugal and 382 from France). The results revealed that most participants were young females (68.9%) with good education (76%) and an average weight range. They consumed a varied diet (57%) but had body dissatisfaction (63.2%). The respondents had good knowledge about the health effects of fruits and vegetables. However, the French population knew more about the theme than the Portuguese. Portuguese individuals were more likely to have incomplete information. Gender and education significantly influenced knowledge levels, with females and highly educated individuals demonstrating greater understanding. Dissatisfaction with body weight drives individuals to seek nutrition information. This investigation enhances our comprehension of the factors that affect knowledge of vegetable and fruit consumption among young adults in Portugal and France. Moreover, it highlights the importance of implementing focused educational programs to enhance nutrition literacy, particularly for less-aware demographic groups. Going forward, a more in-depth analysis of these factors could assist in creating more efficient strategies to encourage healthier dietary habits and improve nutrition literacy among these communities.

## 1. Introduction

Vegetables and fruits are fundamental to a healthy lifestyle, providing diverse vitamins, minerals, phytochemicals, and fiber essential for bolstering the immune system, combatting malnutrition, and preventing noncommunicable disorders across all age groups [1]. However, inadequate diets rank among the top contributors to the global disease burden, emphasizing the significance of fruit and vegetable intake. Regularly consuming these nutrient-dense foods has consistently demonstrated a reduced risk of noncommunicable diseases such as cardiovascular diseases, certain cancers, and obesity [2,3]. Despite their well-established benefits, many individuals fail to reach the recommended daily intake of vegetables and fruits, and research examining this shortfall and its influencing factors remains limited.

The high consumption of plant-based foods, including fruits and vegetables, represents a pivotal feature of Mediterranean dietary patterns, renowned for their myriad health benefits [4,5,6,7,8,9,10]. Shifting current dietary practices towards greater sustainability necessitates understanding the potential for improvement, as augmenting the consumption of fruits, vegetables, and fish may entail sustainability trade-offs [11]. Moreover, dietary diversity forms a cornerstone of a healthy regimen, and optimizing health may involve leveraging local fruits and vegetables [12], with modifications often influenced by social norms and cultural values [13].

Crucially, consumer knowledge concerning the dietary significance of vegetables and fruits plays a pivotal role in influencing their consumption habits. Understanding nutritional value, health advantages, and optimal consumption strategies empowers individuals to make informed dietary choices [14]. Enhancing consumer awareness regarding these dietary impacts is imperative for fostering improved public health outcomes, particularly among younger generations, as early adoption of healthy habits diminishes the likelihood of chronic diseases later in life [15].

Misconceptions regarding portion sizes, preparation techniques, and cooking methods might hinder individuals from maximizing the benefits of these dietary powerhouses [16]. The World Health Organization (WHO) recommends an intake of 400 g of vegetables and fruits (equivalent to 5 servings/day) as a key indicator in its Global Action Plan for the Prevention and Control of NCDs 2013–2020 [17]. Moreover, ensuring the wholesomeness and safety of these food items is paramount, reflected in the increasing domestic and international regulations governing their production, processing, and distribution [17].

Discrepancies in traditional fruit and vegetable consumption often stem from variations in the availability (i.e., quantity) and accessibility (i.e., cost) of imported foods. Higher-income jurisdictions typically boast superior infrastructures facilitating the importation of goods, including food items [18].

Studies such as Mente et al.’s research [19] based on the Prospective Urban Rural Epidemiology (PURE) elucidate fruit and vegetable consumption across diverse populations, offering insights into the influence of dietary patterns on cardiovascular diseases and mortality rates. Additionally, the healthy diet score developed from this study highlights specific foods associated with significantly lower mortality risks, including fruits, vegetables, nuts, legumes, fish, and dairy [19].

In Portugal and France, dietary recommendations align with the latest scientific findings and WHO guidelines [20,21,22]. Despite an appreciation for fresh, flavorful ingredients and traditional culinary methods, both countries exhibit deficiencies in meeting dietary guidelines, particularly in the inadequate consumption of fruits and vegetables [20,22].

This work explores consumers’ understanding of the importance of fruits and vegetables in preventing chronic diseases. Thus, the present research aims to investigate knowledge about the importance of fruits and vegetables in health among populations in Portugal and France. Additionally, it seeks to discern knowledge disparities across diverse demographic factors, encompassing country of origin, gender, education level, body weight satisfaction, varied diet consumption, and chronic diseases.

## 2. Materials and Methods

### 2.1. Instrument and Data Collection

This transversal descriptive study was conducted on individuals 18 years old or above. The survey was carried out in France and Portugal. A questionnaire was developed to gain the necessary information. The questionnaire consisted of 5 parts, where participants self-reported data concerning sociodemographic and anthropometric characteristics. Individuals also answered questions about their perception of current body image, daily behaviors concerning consuming a varied diet and practicing exercise, and health problems (presence of noncommunicable diseases). The fifth and last part evaluated the participants’ knowledge about the benefits of vegetables and fruits through statements that should be answered on a Likert scale from 1 (strongly disagree) to 5 (strongly agree). Two language versions (Portuguese and French) of the survey were used to ensure the questions were understood. Questions in both surveys were the same and followed the same order. 

### 2.2. Participants and Ethics

To be disseminated among participants in Portugal and France, the questionnaire was translated into the two native languages. The online instrument was inserted into the Google Forms^®^ online platform. The questionnaire was disseminated in social networks and by email following a snowball procedure. Data collection took place between December 2020 and May 2021. The exclusion criteria were age under 18 and nationality other than French or Portuguese. The inclusion criteria adopted were: (a) adults (>18 y/o) and (b) residents in Portugal or France, (c) understanding the language, completion of all questions in the survey, contentment to participate in the survey, and agreement to further use of the results in scientific work. Those who wanted to participate accessed the questionnaire items. Those who disagreed were directed to the final page that acknowledged their time. All 639 individuals who attempted to participate in the study, of which 382 were French and 257 were Portuguese, completed the questionnaire and were included.

The research followed the American Psychological Association (APA) Ethical Guidelines for Research involving Human Subjects. The participants were informed about the study’s scope and were not compensated for their participation. Participants gave informed consent when they clicked on ‘Next page’ to start the online survey. The Polytechnic University of Viseu Ethics Committee approved the ethical aspects of this study (No. 10/SUB/2020).

### 2.3. Data Considerations 

For the present study, we made the following considerations about data:

The participants were classified as young adults (between 18 and 25 years old), adults (between 26 and 55 years old), and senior adults (56 years old or over).

From anthropometrically reported data on height (m) and weight (kg), BMI was calculated as weight (kg)/height^2^ (m^2^). 

The participants’ BMI was then classified according to the BMI classes of the World Health Organization (WHO): underweight—BMI < 18.5; average weight—18.5 ≤ BMI < 25; overweight—25 ≤ BMI < 30; obesity—BMI ≥ 30 [23].

The self-reported height and weight provided by the participants have been considered valid, showing moderate to good agreement when compared with direct measurements [24].

Assessment of knowledge: All items used to evaluate knowledge were calculated by the indices as the mean between the participants after reversing the incorrect items (items nº 1, 3, and 7) to place all the items in the same measuring scale, which was redefined to eliminate the effect of the participants who scored 3 (indifferent). Also, each participant’s knowledge level was calculated as the average of all items. In addition, the level of knowledge was divided into the following classes: very low knowledge (−2 ≤ value < −1), low knowledge (−1 ≤ value ≤ 0), high knowledge (0 < value < 1), and very high knowledge (1 ≤ value ≤ 2). 

### 2.4. Statistical Analysis

Descriptive statistical methods, encompassing frequency, mean, and standard deviation analysis, were used for the data analysis. Parametric tests were used for data that presented a normal distribution (according to the Kolmogorov–Smirnov test) [25]. The tests used were the *t*-test for independent samples to compare between two groups and Analysis of Variance—ANOVA, complemented with a post hoc Tukey test to identify the differences between three or more groups [26]. 

In order to ensure that the questionnaire used in the study was reliable and consistent, a Measurement System Analysis (MSA) was performed [27,28,29]. An anti-image matrix was used to assess the MSA values of all 11 items, and it was found that all values were greater than 0.5. This confirms that all 11 items are suitable for inclusion in the analysis.

To evaluate the internal consistency of each factor (F1 [TRUE], F2 [FALSE]), we calculated the Cronbach’s alpha (α) value [30]. The results indicate that factor F1 has a very good level of internal consistency, with a Cronbach’s alpha value of 0.866. In contrast, factor F2 demonstrated good internal consistency with a Cronbach’s alpha value of 0.692 [31,32,33]. Clusters were established after the use of statistical methods [34,35,36].

Finally, the chi-square test assessed differences between clusters according to the sociodemographic factors. The Cramer’s V coefficients were also calculated to measure the association between the categorical variables tested [37].

The software used for the analysis of data was SPSS (Version 28) from IBM Inc. (Armonk, NY, USA), and the level of significance considered was *p* < 0.05.

## 3. Results

### 3.1. Sample Characterization

Table 1 shows the sociodemographic, nutritional status, and behavioral characteristics of the participants and the Portuguese and French groups of participants. Most were young adults, constituting 67.1% of the total sample, with similar percentages in Portugal (70.4%) and France (64.9%). Females comprised a more significant portion, accounting for 68.9% of the total participants, with a higher representation among females in the French sample at 75.9%. Most participants were students, making up 64.9% of the total sample, ranging from 61.0% to 70.8% in France and Portugal, respectively. A significant proportion continued their education to the university level, accounting for 76.4%, with comparable percentages observed in both countries.

Participants generally fell within the normal weight range, although a significant percentage showed overweight (11.9% to 20.6%) or were classified as obese (between 5.0% and 5.4%). A majority (63.2%) expressed dissatisfaction with their body weight, primarily citing excess weight as the primary concern (52.0% in the total sample). The study revealed low levels of physical activity, with a considerable number reporting never or rarely engaging in physical exercise. While most participants reported practicing a varied diet, the frequency varied among individuals. Overall, participants self-reported good to reasonable global health status.

Table 2 provides insight into the prevalence of various health problems among participants from Portugal and France and highlights differences in reported health issues between the two countries and the combined global sample. The prevalence of reported health problems varies between the two countries and the global sample. Obesity emerges as the most prevalent health issue in the global sample, followed by high cholesterol and constipation.

Portugal consistently reported higher occurrences of various health problems, including diabetes, cardiovascular diseases, high cholesterol, constipation, and cancer, in comparison to France. In contrast, France reported a higher incidence of obesity but a lower prevalence of cancer when compared to Portugal.

Many participants in both countries refrained from answering or expressing uncertainty regarding health problems. This trend may reflect uncertainties or privacy concerns among the participants regarding divulging health-related information.

### 3.2. Level of Knowledge about Consumption of Fruits and Vegetables

As explained in the methodology, all eleven items used to assess knowledge were attributed a score. Under these assumptions, the indices calculated for each item are shown in Table 3 as a sum of the scores attributed by all participants and separated for each country. There are noticeable differences in the level of knowledge about fruits and vegetables between Portuguese and French participants, with the French generally demonstrating higher understanding across most aspects examined. Additionally, the global indices portray a better knowledge trend closer to the French participants, indicating a more widespread understanding across various statements among the overall sample than the Portuguese participants.

Table 4 depicts participants’ knowledge regarding fruits and vegetables, analyzing various sociodemographic, anthropometric, and behavioral variables. Differences in knowledge levels were evident between Portugal and France, with participants from France showing notably higher mean knowledge scores than those from Portugal. Gender-based disparities were observed, with females displaying a higher level of knowledge than males, signifying a gender-oriented distinction in understanding the importance of fruits and vegetables. Participants with a university education exhibited significantly higher levels of knowledge than those with a high school education, highlighting an education-related divergence in comprehension. Individuals dissatisfied with their body weight, mainly due to being overweight, showcased higher levels of knowledge compared to those content with their body weight. Consistently, participants reporting a varied diet demonstrated higher knowledge levels than those reporting a less diverse or monotonous diet. There was a noteworthy difference in knowledge among participants with different BMI categories. Individuals categorized as obese showed lower knowledge levels compared to other BMI categories, although this difference was not statistically significant.

### 3.3. Association between Participant Clusters and Sociodemographic Factors Impacting Knowledge of Fruits and Vegetables

The cluster analysis identified three participant clusters: the first comprised individuals with high knowledge of both factors (about the true statements and also about the false statements), the second comprised those with low or very low knowledge (individuals who knew about the true statements and also about the false statements), and the third comprised individuals with partial knowledge, recognizing only the true items and failing to correctly identify the false statements.

After defining the clusters, it is relevant to understand how the different groups, according to sociodemographic, anthropometric, and behavioral factors, distribute among the three clusters. These results are shown in Table 5. These results highlight the distribution of different sociodemographic, anthropometric, and behavioral variables across the identified clusters, indicating significant variations among the clusters in terms of these variables. The percentages represent the prevalence of each class within the respective clusters, and the *p*-values indicate the significance of the association between the variables and cluster membership. A significant difference exists in cluster representation between Portugal and France. France had more participants in Cluster 1, whereas Clusters 2 and 3 were predominantly composed of individuals from Portugal. Age groups and BMI categories did not show significant differences across the clusters, suggesting that age and BMI might not strongly influence knowledge levels regarding fruits and vegetables within these clusters. Gender and education level are significantly linked to cluster membership. Cluster 1 had more females and participants with higher education (university studies), indicating higher knowledge levels in these groups. In contrast males and those with lower education (high school), are more prevalent in Clusters 2 and 3. While not statistically significant, students tended to be more represented in Cluster 1, indicating higher knowledge levels among students. Participants reporting a more frequent varied diet were more present in Clusters 1 and 3, suggesting a possible link between dietary habits and knowledge levels. Moreover, those dissatisfied with their body weight, especially due to being overweight, were more represented in Cluster 1, suggesting a potential association between dissatisfaction and higher knowledge levels about fruits and vegetables.

No significant associations were found between cluster membership and physical exercise frequency or global health status, indicating these factors might not strongly influence knowledge about fruits and vegetables among these clusters.

## 4. Discussion

As far as we know, this is the first study to contrast knowledge regarding fruits and vegetables in France and Portugal. However, it is known that these two nations share analogous food and eating cultures [38]. This paper, involving participants from Portugal and France, reports differences in knowledge regarding the importance of consuming fruits and vegetables in the diet. Furthermore, a relationship between knowledge of fruits and vegetables and sociodemographic and behavioral variables was identified in the studied group. The results provide a comprehensive overview of the sociodemographic characteristics of the study’s participants from Portugal and France. Several key findings can be highlighted.

Most of the participants were young (18–25 years old). The distribution was relatively balanced between the two countries. A higher percentage of female participants was observed in the global sample, with an even higher representation of women in the French sample than in the Portuguese. This prevalence of women has already been documented in other studies [39,40]. Moreover, the respondents presented a high level of education, with a significant proportion of participants identified as students across the global sample, with relatively similar percentages between the French and Portuguese participants. It is worth noting that over three-quarters of participants were either graduates or were attending a university degree course, with consistent percentages across both countries. The demographics of this study are consistent with many trends observed in previous research [41].

A significant proportion of participants reported dissatisfaction with their body weight. This finding is consistent with research by Piryankova and coworkers [42], where many adults expressed dissatisfaction with their body weight and shape. The study further highlights that Portuguese participants exhibited a higher rate of dissatisfaction in comparison to the global sample. The primary reason for this dissatisfaction was excess weight, notably high among the French participants. These data reflect the common concern of weight management and body image among young adults, as explored in the study by Divecha et al. [43]. Despite this dissatisfaction, results indicate a low level of physical exercise among participants, with around one-fifth reporting never engaging in physical activity and about one-third doing it only once a week. This pattern is more pronounced among the French participants.

In the present study, only a quarter of the participants confirmed always following a varied diet. French participants tended to have a higher frequency of practicing a varied diet than the Portuguese. Our results follow those of Entrena-Durán and coworkers [44] regarding the self-perception of healthy eating in Spanish university students. The overall self-reported health status of the participants was generally good, with more than half reporting good health. The Portuguese participants had a slightly higher self-reported reasonable health rate than the French. These results for Portuguese participants are in alignment with data from the Portuguese National Institute of Statistics (INE) publication titled “Income and Living Conditions State of Health 2021”, where individuals with complete secondary or post-secondary education and higher education recorded the highest proportions of positively assessing their health status [45]. It is important to note that a health gap based on educational attainment levels is evident in nearly all EU member states [46,47]. Considering that our respondents were either graduates or attending a university degree course, our results are consistent with this trend. The study noted a relatively low prevalence of major chronic diseases, including cardiovascular diseases, diabetes, obesity, and hypercholesterolemia. However, chronic noncommunicable diseases are one of the most recurrent issues in public health in Portugal [48]. The fact that the participants were young might be responsible for the relatively lower burden of these diseases than in the general population. In contrast, constipation exhibited a higher prevalence, affecting approximately one-tenth of the participants. This finding is of interest, as constipation can be indicative of various factors, including dietary habits, lifestyle, and gastrointestinal health [49].

In the present work, the knowledge about fruits and vegetables was evaluated by a score obtained from the participants’ responses to several statements about the health benefits associated with consuming vegetables and fruits. Globally, all the respondents presented excellent knowledge about health. Results evidence a disparity in the depth of knowledge among participants regarding various health-related aspects of fruits and vegetables. The assessed sample possessed a relatively high level of knowledge concerning the health benefits associated with consuming fruits and vegetables, particularly related to cellular protection and micronutrient content. However, they had a lower level of knowledge regarding the protective effects of certain vegetables against cancer and the potentially irritating effects of some fruits and vegetables on the intestine. This could be because studies linking nutrients and bioactive compounds to health benefits are currently aimed at developing strategies to increase the market polyphenols content in fruits and vegetables for consumers [50]. Considering the respondents were young and emphasized access to the internet to seek information about foods [51], they might attribute more importance to the protective action displayed by the composition of fruits and vegetables than their effects on different diseases, despite these effects being well documented [52,53].

Participants from France exhibited a higher level of knowledge than those from Portugal. France implemented in 2001 the National Nutrition and Health Program (Programme National Nutrition Santé [PNNS]), whose main goal was to improve the health status of the population via nutrition measures [54]. Fruit and vegetables are targeted by one of the nine nutrition priority goals specified in the Programme National Nutrition Santé for 2001–2010 [55]; Portugal has only taken significant steps toward implementing a food and nutrition policy in recent years. The government of Portugal has implemented the Integrated Strategy for the Promotion of Healthy Eating (EIPAS), along with regulatory policies to encourage citizens to make healthier food choices [56]). This has been supported by several communication campaigns and educational materials to raise awareness. However, as documented in the present work, the Portuguese respondents still lack complete knowledge of fruits and vegetables, indicating that there is still work to be done. Despite the promotion of the benefits of fruits and vegetables and their wide availability, the daily consumption of these foods by the adult population in Portugal is still below the recommended levels [56].

Considering that there is a positive link between nutrition knowledge and dietary intake [57], based on our results, we might presume that the French population consumed more fruits and vegetables than the Portuguese. According to EUROSTAT (2019) data, France is one of the four European countries with a higher consumption of vegetables and fruits compared to Portugal. The data shows that 20% of the French population aged 15 years and above consume vegetables and fruits, while only 12% of the Portuguese do so [58]. Even though the Portuguese and French present the same eating culture [59], the French population is more prone to consume vegetables than the Portuguese. Over 60% of the French consumed vegetables at least once daily, against 40% of the Portuguese [60]. Due to a scarcity of epidemiologic studies characterizing, measuring, and analyzing the cultural and environmental imprints in either country, the extent to which these factors impact fruit and vegetable consumption still needs to be seen. This scarcity is primarily due to the need for defined metrics measuring culture accurately. Therefore, until such metrics are identified, our understanding of the influences on vegetable and fruit consumption in the EU remains incomplete. 

Sociodemographic factors have been demonstrated to impact the understanding of the benefits of consuming vegetables and fruits. In our study, female participants exhibited a higher level of knowledge than male participants, confirming sex-related differences in knowledge of fruits and vegetables [41,61]. Women are often associated with concerns about their appearance and tend to adopt a more health-conscious approach to eating [62,63].

Education is another factor impacting the knowledge of fruits and vegetables. Individuals with higher educational attainment had a deeper understanding of the topic, as was found by other studies [64,65]. Consequently, initiatives in public health targeting improved health literacy, particularly among those with lower educational backgrounds, could promote food knowledge and healthier dietary habits in the broader population. 

Participants who were not satisfied with their current body weight and those who believed they were overweight showed a higher level of knowledge. This could indicate that those dissatisfied with their weight were more inclined to seek information about nutrition, including the benefits of consuming fruits and vegetables. However, this is not reflected in the higher consumption of fruits and vegetables, as recently related in a study involving overweight and obese people in the general population between 18 and 65 years old in Türkiye [66]. Previously, a Shelton and coworkers’ study has already related higher BMI to the underconsumption of vegetables in resident adults living in the United States [67]. Research highlights the role of knowledge concerning the capability to eat healthily or enough fruits and vegetables [68]. In our sample, people who know more about vegetables and fruits tend to eat better, adopting a more varied diet. Although this concept may seem obvious, it is not always straightforward and has only been confirmed in some studies. Barbosa and coworkers [69] found that having a high level of nutritional knowledge can impact the adoption of healthy eating habits, but it is not always directly related to body weight.

Findings from our study described herein were in accordance with the main objectives of the present work. Therefore, measures helping consumers obtain nutritional literacy are essential, because those who are more knowledgeable about fruits and vegetables and the health benefits associated with their consumption are significantly more likely to accept an increase in the consumption of these food groups. A study carried out by Speirs et al. showed that adult individuals with a low level of nutritional literacy present a lower predisposition to read food labels and to consume the recommended daily amount of fruits and vegetables [70]. 

Several limitations should be addressed. It is important to note that data collection occurred through online questionnaires, and the absence of the researcher during the respondents’ completion of the questionnaire may have led to uncertainties remaining unaddressed. Additionally, there is a potential for responses to be less authentic, as participants might have opted for what they perceived as the “correct” answer rather than their true sentiments. 

The sample size, although reasonable in number, needs to fully represent young adults, preventing a genuine generalization of the results. Furthermore, there is an imbalance between the group sizes in the two countries, yet both groups exhibited similar trends and had a lower number of adults and older adults. Understandably, interpreting results for these minority groups is inherently limited, precluding generalization—a significant limitation. 

Moreover, because of the specific focus and time constraints of the French study, no validation of the French translation of the questionnaires was performed. Regarding the empirical study framed as a cross-sectional correlational analytical research type, methodological constraints arise due to the evaluations occurring at a single “moment” without a follow-up period for participants. However, this study format offers advantages such as expediency, cost-effectiveness, logistical simplicity, and avoidance of issues associated with loss of follow-up, as seen in longitudinal studies. Despite these limitations, the obtained results contribute to increased knowledge.

Considering all the above, it is time to emphasize the usefulness of the data found in the present study. The findings contribute to the existing literature by providing insights into the variations in knowledge, attitudes, and behaviors related to these dietary components across two culturally similar yet distinct nations. The positive link between nutrition knowledge and dietary intake is highlighted in the study. Individuals with a deeper understanding of fruits and vegetables exhibit better dietary habits, supporting that knowledge influences food choices and consumption patterns. The study reinforces the impact of sociodemographic factors, such as age, gender, and education, on individuals’ knowledge of fruits and vegetables. It aligns with the existing literature highlighting sex-related differences and the influence of education on nutritional knowledge, as extensively explained above. Moreover, some practical consequences might be found, mainly considering country-specific nutrition literacy interventions. As evidenced in the study, Portugal may benefit from initiatives to enhance public understanding of the importance of fruits and vegetables. For instance, developing mobile apps that provide information on the nutritional value, recipes, and benefits of various fruits and vegetables is an idea. It can help people make informed decisions about what they eat and encourage them to incorporate more fruits and vegetables into their diet. Supportive policies like subsidies for fresh produce and community gardens can also effectively promote healthy eating habits. It would be good to see policymakers working towards this goal. Integrating nutritional education into public health programs can also be an excellent way to encourage people to make healthier food choices. Another idea is partnering with companies to incorporate nutritional education and initiatives promoting fruit and vegetable consumption into workplace wellness programs. This measure can help employees make healthier choices, leading to a more productive and healthier workforce. Findings also related dissatisfaction with body weight with higher knowledge levels. Consequently, educational interventions can focus on individuals dissatisfied with their weight, emphasizing the role of a balanced and varied diet in achieving health goals. Additionally, the low percentage of participants reporting following a varied diet indicates room for improvement. Educational campaigns can emphasize the importance of diverse food choices, potentially impacting dietary behaviors. Finally, the study identifies a low level of physical exercise, particularly among the French participants, suggesting the need to incorporate strategies to promote physical activity alongside nutritional education. 

## 5. Conclusions

In short, this pioneering study provides valuable insights into the knowledge disparities regarding fruits and vegetables between France and Portugal, revealing associations with various sociodemographic and behavioral factors. The findings reinforce the need for targeted educational interventions to improve nutrition literacy, especially among lower-awareness demographic groups. To further emphasize the practical implications of our study, particularly for Portugal, we propose country-specific nutrition literacy interventions. For instance, developing mobile apps providing information on nutritional value, recipes, and the benefits of fruits and vegetables can empower individuals to make informed decisions. Supportive policies, such as subsidies for fresh produce and community gardens, can promote healthy eating habits. In light of the dissatisfaction with body weight correlating with higher knowledge levels, we advocate for educational interventions targeting individuals dissatisfied with their weight. Emphasizing the role of a balanced and varied diet in achieving health goals can be pivotal. Additionally, the low percentage of participants reporting a varied diet indicates a need for improvement. We suggest educational campaigns emphasizing diverse food choices to impact dietary behaviors positively. Lastly, the study identifies a low level of physical exercise, particularly among French participants. We recommend incorporating strategies promoting physical activity alongside nutritional education to address lifestyle factors comprehensively.

Despite some data collection and representation limitations, this research helps improve our understanding of factors influencing vegetable and fruit consumption knowledge in young adults from these regions. Moving forward, a deeper exploration of these factors could aid in developing more effective strategies to promote healthier dietary habits and nutrition literacy among diverse populations.

## Figures and Tables

**Table 1:** Sociodemographic, nutritional status, and behavioral characterization of the participants.

Variables	Classes	Portugal [N = 257] n (%) ^1^	France [N = 382] n (%) ^1^	Global [N = 639] n (%) ^1^
Age	Young adults (18–25 y)	181 (70.4)	248 (64.9)	429 (67.1)
	Adults (26–55 y)	65 (25.3)	90 (23.6)	155 (24.3)
	Senior adults (56 y or over)	11 (4.3)	44 (11.5)	55 (8.6)
Sex	Female	150 (58.4)	290 (75.9)	440 (68.9)
	Male	107 (41.6)	92 (24.1)	199 (31.1)
Occupation	Student	182 (70.8)	233 (61.0)	415 (64.9)
	Employed	66 (25.7)	105 (27.5)	171 (26.8)
	Other	9 (3.5)	44 (11.5)	53 (8.3)
Education	High school	61 (23.7)	90 (23.6)	151 (23.6)
	University studies	196 (76.3)	292 (76.4)	488 (76.4)
BMI ^2^	Underweight (BMI < 18.5 kg/m^2^)	37 (15.2)	26 (7.0)	63 (10.2)
	Normal weight (18.5 < BMI ≤ 25.0 kg/m^2^)	165 (67.9)	250 (67.0)	415 (67.4)
	Overweight (25.0 < BMI ≤ 30.0 kg/m^2^)	29 (11.9)	77 (20.6)	106 (17.2)
	Obesity (BMI ≥ 30.0 kg/m^2^)	12 (5.0)	20 (5.4)	32 (5.2)
Satisfaction with body weight	Yes	75 (29.2)	160 (41.9)	235 (36.8)
	No	182 (70.8)	222 (58.1)	434 (63.2)
Reason for dissatisfaction	Low weight	82 (52.9)	38 (23.2)	120 (37.6)
	Overweight	50 (32.3)	116 (70.7)	166 (52.0)
	Obesity	23 (14.8)	10 (6.1)	33 (10.4)
Physical exercise	Never	44 (17.1)	91 (23.8)	135 (21.1)
	Once/week	91 (35.4)	142 (37.2)	233(36.5)
	2–3 times/week	90 (35.0)	100 (26.2)	190 (29.7)
	More than 3 times/week	32 (12.5)	49 (12.8)	81 (12.7)
Varied diet	Never	4 (1.5)	12 (3.1)	16 (2.5)
	Sometimes	128 (49.8)	127 (33.3)	255 (39.9)
	Several times/week	66 (25.7)	146 (38.2)	212 (33.2)
	Always	59 (23.0)	97 (25.4)	156 (24.4)
Global health status	Excellent	13 (5.7)	42 (11.8)	55 (9.4)
	Good	146 (64.0)	204 (57.1)	350 (59.8)
	Reasonable	65 (28.5)	95 (26.6)	160 (27.4)
	Inadequate	4 (1.8)	16 (4.5)	20 (3.4)

^1^ n (%) = number of participants (Percentage); ^2^ BMI = Body Mass Index.

**Table 2:** Number of patients reporting health problems.

Sample		Diabetes n (%)	Obesity n (%)	Cardiovascular Diseases n (%)	Hypercholesterolemia n (%)	Constipation n (%)	Cancer n (%)
Portugal	Yes	18 (7.0)	18 (7.0)	12 (4.7)	23 (8.9)	21 (8.2)	10 (3.9)
	No	228 (88.7)	223 (86.8)	228 (88.7)	215 (83.7)	217 (84.4)	234 (91.0)
	I don’t know	9 (3.5)	13 (7.4)	13 (7.4)	12 (4.7)	11 (4.3)	9 (3.5)
	Don’t want to answer	2 (0.8)	3 (1.2)	4 (1.6)	7 (2.7)	8 (3.11)	4 (1.6)
France	Yes	7 (1.8)	19 (5.0)	7 (1.8)	16 (4.2)	48 (12.6)	2 (0.5)
	No	368 (96.3)	357 (93.5)	363 (95.0)	351 (92.0)	331 (86.7)	371 (97.1)
	I don’t know	5 (1.3)	5 (1.3)	11 (2.9)	13 (3.4)	2 (0.5)	8 (2.0)
	Don’t want to answer	2 (0.5)	1 (0.3)	1 (0.3)	2 (0.5)	1 (0.3)	1 (0.3)
Global	Yes	25 (3.9)	37 (5.8)	19 (3.0)	39 (6.1)	69 (10.8)	12 (1.9)
	No	596 (93.3)	580 (90.8)	591 (92.5)	566 (88.6)	548 (85.8)	605 (94.7)
	I don’t know	14 (2.2)	18 (2.8)	24 (3.8)	25 (3.9)	13 (2.0)	17 (2.7)
	Don’t want to answer	4 (0.6)	4 (0.6)	5 (0,8)	9 (1.4)	9 (1.4)	5 (0.8)

**Table 3:** Indices for knowledge computed for each item based on the sum of scores of the participants.

Item Nº	Statement	Indices for Knowledge		
		Portuguese (N = 257)	French (N = 382)	Global (N = 639)
It. 3	By consuming fruits and vegetables on a daily basis, your body cells will be unprotected (Reversed)	225	521	746
It. 1	Fruits and vegetables are foods poor in vitamins and minerals that are important to the good functioning of the human body (Reversed)	174	552	726
It. 7	Neither vegetables nor fruits contain sugars (Reversed)	153	523	676
It. 2	The regular consumption of vegetables and fruits results in higher and better longevity	210	392	602
It. 8	Both vegetables and fruits constitute a good source of dietary fiber	131	446	577
It. 10	The regular consumption of vegetables and fruits can protect against type 2 diabetes, cardiovascular diseases, hypertension and cancer	159	256	415
It. 11	A high ingestion of fruits and vegetables can reduce obesity, cholesterol, and lower blood pressure	138	265	403
It. 6	Some vegetables such as spinach or cabbage are rich in iron and can help cure anemia	154	215	369
It. 4	Vegetables and fruits are low-calorie foods and can contribute to a healthy weight reduction	77	229	306
It. 5	Green leaves, beans, broccoli, cabbage, cauliflower, and vegetables with a yellow-orange coloration like carrots are more effective in the reduction of cancer incidence	107	65	172
It. 9	Some vegetables and legumes can be irritating to the intestine	−8	126	118

**Table 4:** Knowledge about fruits and vegetables according to sociodemographic, anthropometric, or behavioral variables.

Variables	Classes	Level of Knowledge Mean ^1^ ± SD ^2^	Significance ^3^
Country ^4^	Portugal	0.54 ± 0.71	*p* < 0.001
	France	0.85 ± 0.57	
Age ^5^	Young adults (18–25 y)	0.71 ± 0.63	*p* = 0.600
	Adults (26–55 y)	0.76 ± 0.66	
	Older adults (56 y or over)	0.78 ± 0.75	
Sex ^4^	Female	0.79 ± 0.63	*p* < 0.001
	Male	0.59 ± 0.66	
Occupation ^5^	Student	0.72 ± 0.63	*p* = 0.236
	Employed	0.78 ± 0.68	
	Other	0.62 ± 0.68	
Education ^4^	High school	0.57 ± 0.68	*p* < 0.001
	University studies	0.77 ± 0.63	
BMI ^5^	Underweight (BMI < 18.5 kg/m^2^)	0.66 ± 0.67	*p* = 0.188
	Normal weight (18.5 < BMI ≤ 25.0 kg/m^2^)	0.77 ± 0.61	
	Overweight (25.0 < BMI ≤ 30.0 kg/m^2^)	0.76 ± 0.68	
	Obesity (BMI ≥ 30.0 kg/m^2^)	0.54 ± 0.74	
Satisfaction with body weight ^4^	Yes	0.67 ± 0.67	*p* = 0.004
	No	0.82 ± 0.60	
Reason for Dissatisfaction ^5^	Low weight	0.38 ± 0.64 ^a^	*p* < 0.001
	Overweight	0.82 ± 0.68 ^b^	
	Satisfaction with body weight	0.42 ± 0.74 ^a^	
Physical Exercise ^5^	Never	0.69 ± 0.61	*p* = 0.416
	Once/week	0.71 ± 0.63	
	2–3 times/week	0.73 ± 0.67	
	More than 3 times/week	0.84 ± 0.68	
Varied diet ^5^	Never	0.67 ± 0.59 ^a^	*p* < 0.001
	Sometimes	0.59 ± 0.63 ^a^	
	Several times/week	0.81 ± 0.63 ^b^	
	Always	0.83 ± 0.67 ^b^	
Global Health Status ^5^	Excellent	0.77 ± 0.68	*p* = 0.821
	Good	0.75 ± 0.67	
	Reasonable	0.70 ± 0.58	
	Inadequate	0.67 ± 0.70	
Presence of at least one Chronic Disease ^2^	Yes	0.62 ± 0.76	*p* = 0.052
	No	0.76 ± 0.61	

^1^ Mean = Mean value, ^2^ SD = Standard deviation.^3^ Significance considered in all tests: *p* < 0.05.^4^ *t*-test for independent samples.^5^ ANOVA with post hoc Tukey test. Mean values with different superscript letters are statistically significantly different.

**Table 5:** Association between cluster membership and sociodemographic, anthropometric, and behavioral variables.

Variables	Classes	Cluster 1 % ^1^	Cluster 2 % ^1^	Cluster 3 % ^1^	*p*-Value ^2^	V ^3^
Country	Portugal	40.1	25.3	34.6	<0.001	0.319
	France	71.8	14.1	14.1		
Age	Young adults (18–25 y)	60.6	18.9	20.5	0.418	0.055
	Adults (26–55 y)	56.1	19.4	24.5		
	Senior adults (56 y or over)	54.5	14.6	30.9		
Sex	Female	64.1	16.1	19.8	<0.001	0.154
	Male	47.7	24.1	28.2		
Occupation	Student	61.4	18.3	20.3	0.189	0.069
	Employed	57.3	18.1	24.6		
	Other	45.3	22.6	32.1		
Education	High school	43.0	26.5	30.5	<0.001	0.181
	University studies	63.9	16.2	19.9		
BMI ^4^	Underweight (BMI < 18.5 kg/m^2^)	57.1	20.6	22.2	0.400	0.071
	Normal weight (18.5 < BMI ≤ 25.0 kg/m^2^)	62.4	16.4	21.2		
	Overweight (25.0 < BMI ≤ 30.0 kg/m^2^)	53.8	17.9	28.3		
	Obesity (BMI ≥ 30.0 kg/m^2^)	53.1	28.1	18.8		
Satisfaction with body weight	Yes	55.2	20.3	24.5	0.038	0.101
	No	65.6	15.7	18.7		
Physical Exercise	Never	62.2	21.5	16.3	0.205	0.080
	Once/week	58.4	15.9	25.7		
	2–3 times/week	54.7	21.6	23.7		
	More than 3 times/week	65.4	14.8	19.8		
Varied diet	Never	50.0	31.3	18.8	0.001	0.131
	Sometimes	49.0	22.0	29.0		
	Several times/week	68.4	16.0	15.6		
	Always	63.5	15.4	21.2		
Global health Status	Excellent	67.3	20.0	12.7	0.408	0.072
	Good	59.4	16.6	24.0		
	Reasonable	55.6	22.5	21.9		
	Inadequate	65.0	15.0	20.0		

^1^ Percentage in line; ^2^ Significance of the chi-square test (*p* < 0.05); ^3^ Cramer’s coefficient; ^4^ BMI = body mass index.

## Data Availability

Data are available from the corresponding author, upon reasonable request. The data are not publicly available due to privacy.
